# Trajectories of processing speed in multiple sclerosis across disease-modifying therapies

**DOI:** 10.1007/s00415-026-13860-8

**Published:** 2026-05-20

**Authors:** Jie Guo, Eva Johansson, Jan Hillert, Tomas Olsson, Lars Alfredsson, Anna Karin Hedström

**Affiliations:** 1 Department of Nutrition and Health, China Agricultural University, Beijing, China; 2 Department of Clinical Neuroscience, Karolinska Institutet, Stockholm, Sweden; 3 Institute of Environmental Medicine, Karolinska Institutet, Stockholm, Sweden; 4 Centre for Occupational and Environmental Medicine, Region Stockholm, Stockholm, Sweden

**Keywords:** Multiple sclerosis, processing speed, cognitive function, disease-modifying therapy, longitudinal study

## Abstract

**Background:**

Cognitive impairment is a common feature of multiple sclerosis (MS), affecting daily functioning and quality of life. We aimed to investigate how disease-modifying therapies (DMTs) of varying efficacy are associated with long-term cognitive trajectories in MS, as measured by the Symbol Digit Modalities Test (SDMT).

**Methods:**

Using the data from Swedish population-based MS cohorts linked to the Swedish MS registry, we included individuals with relapsing-onset MS receiving DMT to evaluate longitudinal SDMT trajectories. From a cohort of 5467 treated individuals, several analytic subsets were defined for specific trajectory analyses. Mixed-effects models were used to examine changes in cognitive performance across treatment categories, including platform therapies, high-efficacy therapies overall, and specific high-efficacy agents.

**Results:**

SDMT scores increased during the first years of follow-up among individuals initiating high-efficacy DMT and subsequently declined, whereas changes in the platform group were smaller over time. Differences between groups were observed at years 3 and 4, with an estimated difference of approximately 2 points, which were not sustained at later follow-up. When compared with anti-CD20 therapies, natalizumab-treated individuals showed a greater early increase (*β* − 0.77, 95% CI − 1.26, − 0.27) and a more pronounced subsequent decline (*β* 0.38, 95% CI 0.21–0.55), with trajectories converging over time. Adjustment for repeated testing attenuated early increases in SDMT scores.

**Conclusions:**

There was limited evidence for a sustained or clinically relevant short-term effect of high-efficacy DMTs on cognitive trajectories in MS. Differences between groups and changes over time are likely influenced by underlying patient characteristics and practice effects related to repeated testing.

**Supplementary Information:**

The online version contains supplementary material available at 10.1007/s00415-026-13860-8.

## Introduction

Multiple sclerosis (MS) is a chronic, demyelinating disorder of the central nervous system, characterized by inflammation, progressive neuroaxonal degeneration, and cerebral gray matter atrophy [1, 2]. Cognitive impairment is a common feature in MS [3, 4], affecting several cognitive domains and often co-occurring with fatigue and depression [5, 6]. Although more pronounced in progressive phases of MS [7, 8], cognitive deficits can emerge early and tend to worsen over time, often in parallel with cerebral atrophy [9–13].

Despite its prevalence, evidence regarding the efficacy of disease-modifying therapies (DMTs) on cognitive impairment remains limited. Meta-analyses have suggested modest benefits of DMTs on cognitive performance, but no significant differences have been found between specific agents, such as β-interferon and other substances [14]. A major challenge in studies of treatment effects is that patients receiving high-efficacy DMTs typically have more aggressive disease, making it difficult to disentangle treatment effects from disease severity. Most earlier studies have been restricted to relatively short follow-up periods, limiting insights into whether potential cognitive improvements are sustained over time. Furthermore, repeated cognitive testing may introduce practice effects [15–17], which can contribute to apparent improvements in performance over time and complicate the interpretation of longitudinal changes.

The primary aim of this study was to compare longitudinal Symbol Digit Modalities Test (SDMT) trajectories between patients initiating platform versus high-efficacy DMTs. We further aimed to examine SDMT trajectories by time from disease onset to initiation of high-efficacy therapy, differences between specific high-efficacy agents (natalizumab vs anti-CD20 therapies), and changes in SDMT trajectories before and after escalation from platform to high-efficacy treatment.

## Methods

Our study included patients from Epidemiologic Investigation of Multiple Sclerosis (EIMS) [18], Genes and Environment in Multiple Sclerosis (GEMS) [18] and Immunomodulation and Multiple Sclerosis Epidemiology study (IMSE) [19].

EIMS and GEMS are population-based case–control studies with a study base comprising the Swedish population aged 16–70 years. EIMS recruited incident cases from neurology units at hospitals up to December 2019 (*n* = 3558), whereas GEMS identified prevalent cases from the Swedish MS registry between November 2009 and November 2011 (*n* = 6136). IMSE is a cohort study including MS patients initiating different DMTs, designed to evaluate how demographic and clinical factors influence treatment response (*n* = 1009). The response rates were 93% for EIMS, 82% for GEMS, and 68% for IMSE, respectively. All participants met the McDonald criteria for MS [20]. The study populations were mutually exclusive, with no participants appearing in more than one study.

Of the 10,703 total patients, we excluded those with progressive-onset MS (*n* = 832), those who had never received DMTs (*n* = 1597), those who discontinued treatment before SDMT was introduced in clinical practice (*n* = 587), and those without SDMT assessments during their initial treatment (*n* = 2220), resulting in a source population of 5467 patients (eFigure 1). From this source population, several analytic samples were defined for different research questions (Table 1).

**Table 1:** Overview of analytic cohorts and time-zero definitions

Analysis	Population definition	Time-zero	Sample size
Primary analysis	DMT-naïve participants initiating first-ever DMT (platform or HE-DMT)	DMT initiation	*n* = 1827
Timing of HE-DMT initiation	All participants initiating first HE-DMT	DMT initiation	*n* = 3431
Therapy comparison	Participants initiating natalizumab or anti-CD20 therapy	DMT initiation	*n* = 727
Escalation analysis	Participants treated with platform therapy, with or without subsequent switch to HE-DMT	Time of switch (T0) or corresponding time in nonswitchers	*n* = 3601

All analyses were conducted within the same underlying cohort of relapsing-onset MS patients (*n* = 5467), but with analysis-specific inclusion criteria and definitions of time-zero. Consequently, the analytic samples are not mutually exclusive. The therapy comparison analysis (natalizumab vs anti-CD20) is nested within the group of participants initiating HE-DMT

*HE-DMT* high-efficacy disease-modifying therapy

### Definition of exposures and outcome measures

The Swedish MS registry, utilized by neurology units nationwide, serves as an integrated system within clinical documentation, where comprehensive information for each patient is continuously recorded [21]. Physicians and nurses enter data on medical treatment, disease activity, physical functioning, mental health, cognitive performance, and quality of life.

The exposure was DMT. High-efficacy DMTs comprised natalizumab, fingolimod, ocrelizumab, alemtuzumab, cladribine, mitoxantrone, and rituximab. Platform treatment included interferon-beta, glatiramer acetate, teriflunomide, and dimethyl fumarate.

Cognitive performance was assessed using the SDMT, which measures processing speed and attention by asking participants to match symbols to numbers within a set timeframe. SDMT is recognized as a standard longitudinal outcome measure in MS [22, 23]. Baseline SDMT scores were defined as the scores recorded closest to the initiation of DMT. If no pre-treatment assessment was available, the first post-initiation SDMT was used. In Swedish clinical practice, SDMT is typically administered in its standard written format as part of routine neurological assessments. Information on the use of alternate test forms was not available, however, testing procedures are generally standardized across clinics.

### Statistical analysis

Categorical variables were summarized using frequency and percentage, whereas continuous variables were summarized using mean and standard deviation (SD). Longitudinal changes in SDMT scores were analyzed using mixed-effect models with random intercepts and random slopes for time. The general model included linear and quadratic terms for time, treatment group, and their interactions:

$${\mathrm{SDMT}}_{{{\mathrm{ij}}}} \, = \,\beta_{0} \, + \,\beta_{{1}} {\mathrm{Time}}_{ij} \, + \,\beta_{{2}} {\mathrm{Time}}_{ij}^{{2}} \, + \,\beta_{{3}} {\mathrm{Group}}_{i} \, + \,\beta_{{4}} \left( {{\mathrm{Time}}_{ij} \, \times \,{\mathrm{Group}}_{i} } \right)\, + \,\beta_{{5}} \left( {{\mathrm{Time}}_{ij}^{{2}} \, \times \,{\mathrm{Group}}_{i} } \right)\, + \,X_{i} \gamma \, + \,b_{0i} \, + \,b_{1i} {\mathrm{Time}}_{ij} \, + \,\varepsilon_{ij} .$$

Here, $i$ ndexes individuals and $j$ measurement occasions; ${group}_{i}$ denotes the treatment category; ${X}_{i}$ represents baseline covariates, including the number of repeated SDMT assessments as a proxy for retesting; and ${b}_{0i}$ and ${b}_{1i}$ are individual-level random effects. Model diagnostics indicated good overall fit. All analyses were restricted to participants with at least two SDMT measurements within the relevant treatment period to enable within-individual change over time. The proportion of participants excluded due to fewer than two SDMT measurements was similar between treatment groups.

The SDMT was introduced into clinical MS care in 2006. The primary analysis included DMT-naïve patients who initiated treatment with either platform or high-efficacy DMTs, grouped according to their initial DMT category (*n* = 1827). Platform therapy served as the reference. SDMT trajectories were modeled although the patients remained on treatment within their original efficacy category, ensuring trajectories under treatment exposure. For multiple comparisons of estimated SDMT values, a simulation-based step-down approach was used to calculate adjusted *P* values and CIs [24].

We subsequently analyzed patients who initiated their first treatment with high-efficacy DMT (*n* = 3431). To examine whether timing influenced cognitive outcomes, patients were grouped according to the interval from disease onset to initiation of high-efficacy DMT (< 5 years, 5–10 years, or > 10 years). Within the high-efficacy group, SDMT trajectories were also compared separately for patients treated with natalizumab and those treated with anti-CD20 therapies (*n* = 727).

A separate analysis focused on patients who switched from platform to high-efficacy therapy during follow-up (*n* = 3601). For this subgroup, the time of the switch was defined as time 0. SDMT scores were analyzed over the 5 years prior to and 8 years after the switch. SDMT scores after discontinuation of high-efficacy DMTs among switchers were not considered, nor were SDMT scores after cessation of platform DMTs among nonswitchers. Two distinct trajectories were plotted: one representing those who remained on platform therapy until time 0, and another for those who transitioned to high-efficacy therapy at time 0. This allowed us to visualize cognitive changes associated with treatment escalation.

All analyses were adjusted for sex, educational attainment (university education, no university education, or unknown), number of repeated SDMT measurements, time between first clinical symptoms of disease (disease onset) and DMT initiation (or treatment transition), and age at DMT initiation or age at transitioning to high-efficacy DMT. The number of repeated SDMT measurements was included as a proxy for cumulative test exposure to account for potential practice effects.

In a sensitivity analysis, SDMT trajectories were stratified by baseline SDMT (above vs below the median) to assess whether baseline cognitive performance influenced longitudinal patterns. We performed sensitivity analyses restricted to incident MS cases and further adjusted for ancestry (Nordic or non-Nordic), smoking (current vs nonsmokers), alcohol consumption (yes vs no), body mass index (< 25, 25–30, > 30 kg/m^2^), past infectious mononucleosis (yes, no, unsure), sun exposure, regular physical activity, baseline Expanded Disability Status Scale (EDSS) score, and Multiple Sclerosis Impact Scale (MSIS-29) psychological and mental component. Physical activity was classified as low or high, with high activity defined as exercising at least 30 min at least twice per week at an intensity sufficient to induce sweating. Sun exposure was classified as low or high based on a composite measure of sunbathing habits, travel to sunny destinations, and sunbed use. All analyses were conducted in SAS 9.4 (SAS Institute, Cary, NC, USA).

## Results

From the full cohort of 5467 patients with relapsing-onset MS who initiated either platform or high-efficacy DMTs, several analytic subsets were defined for specific trajectory analyses (eTable 1). Baseline characteristics for the full cohort are presented in Table 2. Mean age at disease onset was 31.6 years (SD 9.7) and mean age at treatment initiation was 36.3 years (SD 10.3). Additional baseline characteristics for the analytic samples included in each of the SDMT trajectory analyses are presented in eTables 1–4. Across analyses, participants initiating high-efficacy DMT had lower baseline SDMT scores, higher EDSS scores, and more frequent SDMT assessments than those initiating platform therapy.

**Table 2:** Baseline characteristics of participants

Characteristic	Total (*n* = 5467)
Age at disease onset, years (SD)	31.6 ± 9.7
University education, *n* (%)	
Yes	2073 (37.9)
No	2601 (47.6)
Missing	793 (14.5)
Sex, *n* (%)	
Female	3979 (72.8)
Male	1488 (27.2)
Ancestry, *n* (%)	
Nordic	4535 (83.5)
Non-Nordic	894 (16.5)
Initial DMT efficacy class, *n* (%)	
Platform	4554 (83.3)
High-efficacy	913 (16.7)
Age at DMT initiation, years (SD)	36.3 (10.3)
Duration between onset and DMT initiation, years (SD)	4.7 (6.4)
Mean baseline EDSS (SD)	2.5 (1.7)
Mean baseline SDMT (SD)	51.2 (12.8)

*DMT* disease-modifying therapy, *EDSS* Expanded Disability Status Scale, *SDMT* Symbol Digit Modalities Test, *SD* standard deviation

**Fig. 1:**
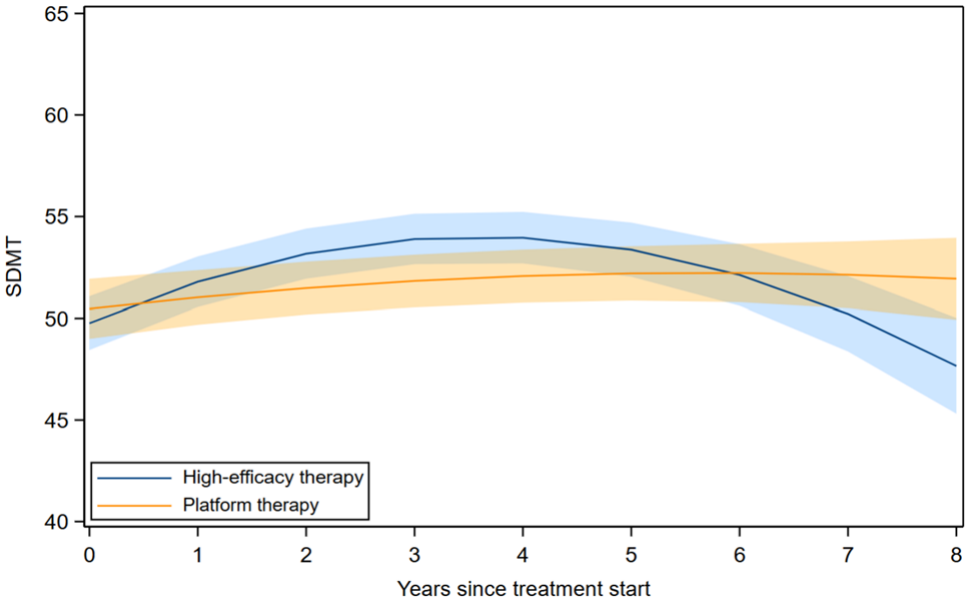
SDMT trajectories among DMT-naïve participants initiating platform versus high-efficacy DMT. *SDMT* Symbol Digit Modalities Test, *DMT* high-efficacy disease-modifying therapy; adjusted for age at DMT initiation, sex, education, the number of repeated SDMT measurements, and the duration between disease onset and DMT initiation. The bands represent the 95% confidence interval for the estimated mean SDMT

In the analysis of DMT-naïve participants initiating first treatment, SDMT scores initially increased in the high-efficacy group, whereas changes in the platform group were smaller over time (Fig. 1, eTable 5). Estimated mean SDMT scores were higher in the high-efficacy group at years 3 and 4 (eTable 6). Thereafter, differences between groups decreased, and by year 8, the estimated mean SDMT was higher in the platform group (Fig. 1, eTable 5).

**Fig. 2:**
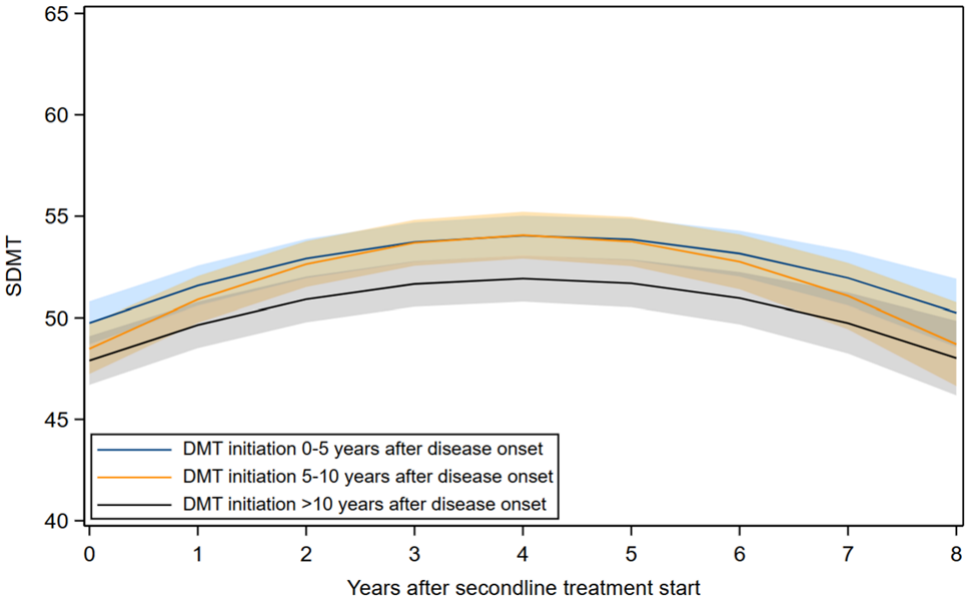
SDMT trajectories after high-efficacy DMT initiation, stratified by time from disease onset to DMT initiation. *SDMT* Symbol Digit Modalities Test, *DMT* high-efficacy disease-modifying therapy; adjusted for age at DMT initiation, sex, education, the number of repeated SDMT measurements, and time between disease onset and DMT initiation. The bands represent the 95% confidence intervals for the estimated mean SDMT

Among participants initiating high-efficacy DMT, baseline SDMT scores differed by time from disease onset to treatment initiation. There were no significant differences in changes in SDMT scores over time between the timing groups (Fig. 2, eTable 7).

**Fig. 3:**
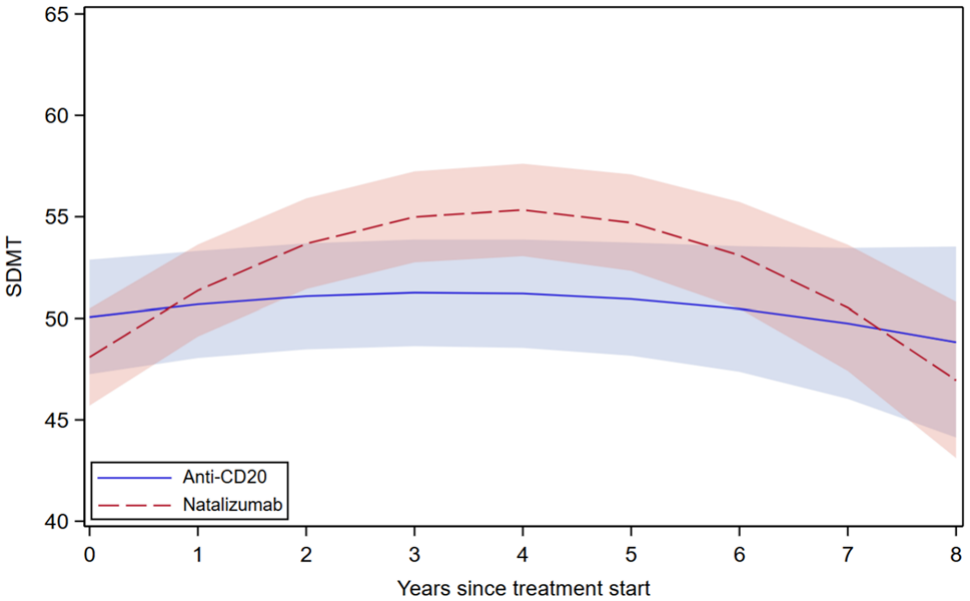
SDMT trajectories after initiation of Natalizumab versus anti-CD20 therapies. *SDMT* Symbol Digit Modalities Test, *DMT* high-efficacy disease-modifying therapy; adjusted for age at DMT initiation, sex, education, the number of repeated SDMT measurements, and time between disease onset and DMT initiation. The bands represent the 95% confidence intervals for the estimated mean SDMT

When comparing individual high-efficacy therapies, SDMT scores showed a greater initial increase and subsequently a more pronounced decline in participants treated with natalizumab than in those receiving anti-CD20 therapies. At later follow-up, the trajectories approached similar SDMT levels (Fig. 3, eTable 8).

**Fig. 4:**
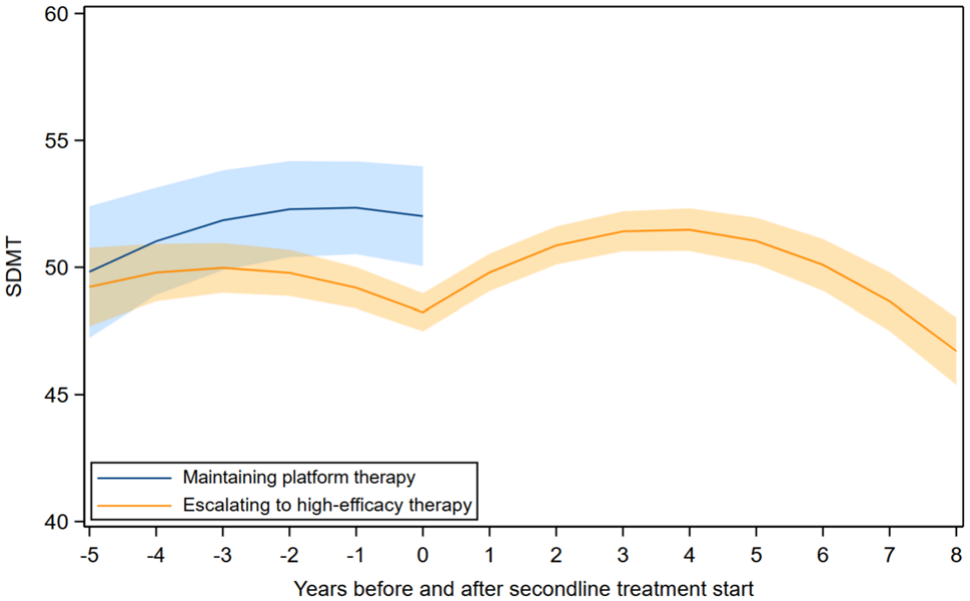
SDMT trajectories for participants who initiated platform therapy, shown separately for those who escalated to high-efficacy DMT and those who remained on platform therapy. *SDMT* Symbol Digit Modalities Test, *DMT* high-efficacy disease-modifying therapy. Adjusted for age at year 0, sex, education, the number of repeated SDMT measurements, and time between disease onset and year 0. Year 0 represents the time of escalation to high-efficacy therapy. The bands represent the 95% confidence intervals for the estimated mean SDMT

Before transition from platform to high-efficacy DMT, there were no significant differences in SDMT trajectories between participants who later switched and those who remained on platform therapy. After transition, SDMT scores increased in the switching group during the first years of follow-up, followed by a subsequent decline (Fig. 4, eTable 9).

Participants with baseline SDMT above median had higher SDMT scores throughout follow-up, while showing a smaller increase over time compared with those below the median. Both trajectories showed a decline at later follow-up (eFigure 3, eTable 10).

In analyses restricted to incident cases and additionally adjusted for lifestyle-related covariates, the primary comparison between platform and high-efficacy DMT was attenuated, whereas the overall pattern for natalizumab versus anti-CD20 therapies was broadly similar, although some interaction estimates were weakened (eFigure 2–3, eTables 11–12).

## Discussion

We investigated long-term SDMT trajectories in patients with relapsing-onset MS initiating platform versus high-efficacy DMTs. SDMT trajectories in the high-efficacy group were characterized by an initial increase followed by a subsequent decline, whereas changes in the platform group were smaller and more stable over time. Notably, trajectories among natalizumab-treated patients differed from those receiving anti-CD20 therapies, with a greater early increase followed by a more pronounced subsequent decline, converging towards similar levels at later follow-up.

The early increase in SDMT scores among participants initiating high-efficacy therapy should be interpreted with caution, as adjustment for repeated testing substantially attenuated these effects, suggesting that practice effects may account for a substantial part of the observed changes. As practice effects plateau over time [15–17], subsequent changes may more closely reflect underlying disease processes. In addition, participants initiating high-efficacy therapy had lower SDMT scores and higher disability at treatment start than those initiating platform therapy, consistent with treatment allocation based on disease severity, which may contribute to regression to the mean. Although small increases in SDMT may partly reflect recovery from inflammation-related dysfunction [25, 26], these do not appear to translate into clinically relevant differences in cognitive trajectories.

When patients on high-efficacy therapies were categorized according to time from disease onset to DMT initiation, baseline SDMT scores differed between groups, whereas no differences in SDMT trajectories over time were observed, suggesting that timing of treatment initiation had no clear influence on longitudinal changes in SDMT performance.

Natalizumab-treated individuals showed a greater initial increase in SDMT scores followed by a more pronounced subsequent decline compared with those treated with anti-CD20 therapies, with trajectories converging towards similar levels at later follow-up. These differences may partly reflect differences in testing frequency and patient characteristics between groups, which may influence both the magnitude of early changes and the shape of trajectories over time.

Our analysis of patients who switched from low- to high-efficacy treatment revealed no significant differences in SDMT trajectories prior to treatment transition. Following transition, SDMT scores increased initially within the switching group, followed by a subsequent decline over time. Given the absence of clear between-group differences and the potential influence of repeated testing and other nonspecific effects, these findings should be interpreted with caution.

Previous studies have reported mixed findings regarding the effects of DMTs on cognitive outcomes in MS. Overall, evidence from randomized trials has been limited [25] and observational studies have suggested modest improvements in cognitive performance without clear differences between treatment classes [14]. Our findings align with this pattern and provide limited evidence for sustained differences between treatment groups and indicate that observed early changes may partly reflect measurement-related effects.

A key strength of this study is its longitudinal design with extended follow-up, enabling detailed characterization of cognitive trajectories over time. The use of real-world clinical data enhances the generalizability of the findings. Adjustment for relevant confounders and repeated SDMT measurements increases the robustness of the results, with the latter allowing partial accounting for practice effects, which materially influenced the observed findings.

Despite its strengths, this study has several limitations. Patients prescribed high-efficacy treatments may have more active disease, potentially biasing comparisons across treatment groups. Restriction to on-treatment periods, including censoring at treatment discontinuation or switch, may introduce informative censoring, as treatment changes are not random. Baseline SDMT was defined as the measurement closest to treatment initiation and in some cases occurred after treatment start, which may have introduced early treatment effects into baseline values and may attenuate estimates of early changes over time. Relapse activity and recent corticosteroid use at the time of SDMT assessment were not accounted for, potentially introducing additional variability in SDMT scores. Adjustment for repeated SDMT assessments was used as a proxy for cumulative test exposure but does not fully capture practice effects, which may still influence longitudinal changes. Residual confounding, including mental health status and unmeasured comorbidities, cannot be excluded.

In conclusion, high-efficacy DMTs were associated with modest differences in SDMT trajectories compared with platform therapies, with no clear evidence of sustained cognitive benefit over time. Early increases in SDMT scores were substantially attenuated after accounting for repeated testing, suggesting that practice effects contribute to these changes. Overall, the findings provide limited evidence for a clinically meaningful or durable effect of high-efficacy DMTs on cognitive trajectories as measured by SDMT.

## Supplementary Information

Below is the link to the electronic supplementary material.

Supplementary file1 (DOCX 296 KB)

Supplementary file2 (DOCX 33 KB)

## Data Availability

Anonymized data underlying this article will be shared on reasonable request from any qualified investigator that wants to analyze questions that are related to the published article.
