# Supplementary material for: Trajectories of processing speed in multiple sclerosis across disease-modifying therapies
Source: J Neurol. 2026 May 20;273(6):324. doi: 10.1007/s00415-026-13860-8 (PMC13190381; doi:10.1007/s00415-026-13860-8)
Supplement: Supplementary file 1 — Supplementary file1 (DOCX 296 KB) [file 415_2026_13860_MOESM1_ESM.docx]

**Supplementary files**

eTable 1. Baseline characteristics of participants included in the comparison of SDMT trajectories between those who initiated platform versus high-efficacy DMT.

eTable 2. Baseline characteristics of participants included in the analysis of SDMT trajectories by timing of high-efficacy DMT initiation.

eTable 3. Baseline characteristics of participants included in the comparison of SDMT trajectories between natalizumab an anti-CD20 therapies.

eTable 4. Baseline characteristics of participants included in the analysis of SDMT trajectories before and after escalation from platform to high-efficacy therapy.

eTable 5. β coefficients and 95% CIs for SDMT trajectories over eight years after DMT initiation, by efficacy category.

eTable 6. Differences in SDMT scores between participants initiating platform versus high-efficacy DMT.

eTable 7. β coefficient and 95% CIs for SDMT trajectories over eight years, by time from disease onset to high-efficacy DMT initiation.

eTable 8. β coefficient and 95% CIs for SDMT trajectories over eight years among participants initiating Natalizumab versus anti-CD20 therapy.

eTable 9. β coefficients and 95% CIs for SDMT trajectories before and after initiation of high-efficacy DMT, comparing participants who escalated from platform to high-efficacy DMT with those who remained on platform therapy.

eTable 10. β coefficients and 95% CIs for SDMT trajectories among participants initiating high-efficacy DMT initiation, by baseline SDMT score (<median vs >median)

eTable 11. Sensitivity analysis limited to incident cases: β coefficients and 95% CIs for SDMT trajectories over eight years after DMT initiation, by efficacy category.

eTable 12. Sensitivity analysis limited to incident cases: β coefficient and 95% CIs for SDMT trajectories over eight years among participants initiating Natalizumab versus anti-CD20 therapy.

eFigure 1. Flow chart of the study population

eFigure 2. SDMT trajectories among participants initiating high-efficacy DMT, by baseline SDMT score (<median vs >median)

eFigure 3. SDMT trajectories among DMT-naïve participants initiating platform versus high-efficacy DMT (incident cases)

eFigure 4. SDMT trajectories after initiation of Natalizumab versus anti-CD20 therapies (incident cases)

eTable 1. Baseline characteristics of participants included in the comparison of SDMT trajectories between those who initiated platform versus high-efficacy DMT.

| Characteristic | | Platform therapy | High-efficacy DMT | P value |
| --- | --- | --- | --- | --- |
| N | | 989 | 838 |  |
| N of repeated SDMT measurements, median (25^th^, 75^th^) | | 3 (2,5) | 6 (4,9) | <0.001 |
| Median interval between SDMT measurements, years (25^th^, 75^th^) | | 1.0 (0.7, 1.3) | 0.7 (0.5, 1.0) | <0.001 |
| Frequency of SDMT measurements/year | | 0.4 (0.3, 0.5) | 0.8 (0.5, 1.0) | <0.001 |
| Median follow-up time, years (25^th^, 75^th^) | | 8.0 (7.2,8.0) | 7.9 (5.3, 8.0) | <0.001 |
| Age at disease onset, years (SD) | | 33.4 (10.4) | 32.4 (10.1) | 0.06 |
| Age at DMT initiation, years (SD) | | 37.4 (10.5) | 36.6 (11.4) | 0.11 |
| University, n (%) | Yes | 425 (43.0) | 345 (41.2) | 0.52 |
|  | No | 510 (51.6) | 441 (52.6) |  |
|  | Missing | 54 (5.5) | 52 (6.2) |  |
| Years between onset and DMT initiation (SD) | | 3.9 (6.2) | 4.8 (7.1) | 0.01 |
| Months between baseline SDMT and DMT initiation (SD) | | 1.1 (2.2) | 1.0 (2.1) | 0.37 |
| Sex (%) | Female | 729 (73.7) | 586 (69.9) | 0.07 |
|  | Male | 260 (26.3) | 252 (30.1) |  |
| Ancestry (%) | Nordic | 805 (82.4) | 671 (80.2) | 0.22 |
|  | Non-Nordic | 172 (17.6) | 166 (19.8) |  |
| Mean baseline EDSS (SD) | | 1.6 (1.3) | 2.2 (1.7) | <0.001 |
| Mean SDMT score at DMT initiation (SD) | | 53.3 (12.5) | 50.8 (12.5) | 0.001 |

SDMT=Symbol Digit Modalities Test; DMT=disease-modifying therapy; EDSS=Expanded Disability Status Scale; SD=standard deviation.

eTable 2. Baseline characteristics of participants included in the analysis of SDMT trajectories by timing of high-efficacy DMT initiation.

| Characteristic | | Time between MS onset and DMT initiation | | | P value |
| --- | --- | --- | --- | --- | --- |
|  |  | 0-5 | 5-10 | >10 |  |
| N | | 1443 | 761 | 1227 |  |
| N of repeated SDMT measurements, median (25^th^, 75^th^) | | 7 (4, 11) | 7 (4, 11) | 6 (4, 9) | <0.001 |
| Median interval between SDMT measurements, years (25^th^, 75^th^) | | 0.66 (0.50, 0.96) | 0.67 (0.51, 0.96) | 0.74 (0.52, 1.02) | <0.001 |
| Frequency of SDMT measurements/year | | 1.1 (0.8, 1.6) | 1.0 (0.7, 1.5) | 1.0 (0.6, 1.4) | <0.001 |
| Median follow-up time, years (25^th^, 75^th^) | | 8.00 (5.81, 8.00) | 8.00 (6.22, 8.00) | 8.00 (5.42, 8.00) | <0.001 |
| Age at disease onset, years (SD) | | 32.6±9.9 | 31.6±9.0 | 28.4±8.7 | <0.001 |
| Age at DMT initiation, years (SD) | | 34.9±10.0 | 39.5±9.1 | 46.2±9.1 | <0.001 |
| Months between baseline SDMT and DMT initiation (SD) | | 1.2 (2.2) | 1.3 (2.4) | 1.4 (2.5) | 0.0618 |
| Years between onset and DMT initiation (SD) | | 2.3±1.6 | 7.9±1.4 | 17.8±6.3 | <0.001 |
| University, n (%) | Yes | 630 (43.7) | 308 (40.5) | 340 (27.7) | 0.82 |
|  | No | 785 (54.4) | 402 (52.8) | 417 (34.0) |  |
|  | Missing | 28 (1.9) | 51 (6.7) | 470 (38.3) |  |
| Sex (%) | Female | 1000 (69.3) | 564 (74.1) | 916 (74.7) | 0.002 |
|  | Male | 443 (30.7) | 197 (25.9) | 311 (25.3) |  |
| Ancestry (%) | Nordic | 1136 (79.1) | 612 (80.7) | 1064 (86.9) | <0.001 |
|  | Non-Nordic | 300 (20.9) | 146 (19.3) | 161 (13.1) |  |
| Mean baseline EDSS (SD) | | 2.1±1.6 | 2.3±1.7 | 3.1±1.9 | <0.001 |
| Mean SDMT score at DMT initiation (SD) | | 52.6±12.2 | 50.4±11.9 | 47.3±12.6 | <0.001 |

SDMT=Symbol Digit Modalities Test; DMT=disease-modifying therapy; EDSS=Expanded Disability Status Scale; SD=standard deviation.

eTable 3. Baseline characteristics of participants included in the comparison of SDMT trajectories between those who initiated natalizumab versus anti-CD20 therapies.

| Characteristic | | Natalizumab | Anti-CD20 | P value |
| --- | --- | --- | --- | --- |
| N | | 402 | 325 |  |
| N of repeated SDMT measurements, median (25^th^, 75^th^) | | 8 (5, 13) | 4 (3, 6) | <0.001 |
| Median interval between SDMT measurements, years (25^th^, 75^th^) | | 0.5 (0.4,0.7) | 1.0 (0.8,1.2) | <0.001 |
| Frequency of SDMT measurements/year | | 1.1 (0.6, 1.8) | 0.9 (0.7, 1.2) | <0.001 |
| Median follow-up time, years (25^th^, 75^th^) | | 8.0 (7.8,8.0) | 5.4 (3.9,7.1) | <0.001 |
| Age at disease onset, years (SD) | | 30.9±9.2 | 33.7±11.0 | <0.001 |
| Age at DMT initiation, years (SD) | | 33.7±10.3 | 40.0±12.1 | <0.001 |
| Months between baseline SDMT and DMT initiation (SD) | | 0.9±2.0 | 1.0±2.2 | 0.701 |
| Years between onset and DMT initiation (SD) | | 3.7±5.9 | 5.6±8.1 | <0.001 |
| University, n (%) | Yes | 165 (41.0) | 137 (42.2) | 0.87 |
|  | No | 210 (52.2) | 170 (52.3) |  |
|  | Missing | 27 (6.7) | 18 (5.5) |  |
| Sex (%) | Female | 292 (72.6) | 220 (67.7) | 0.146 |
|  | Male | 110 (27.4) | 105 (32.3) |  |
| Ancestry (%) | Nordic | 316 (78.8) | 270 (83.1) | 0.147 |
|  | Non-Nordic | 85 (21.2) | 55 (16.9) |  |
| Mean baseline EDSS (SD) | | 2.3±1.7 | 2.0±1.6 | 0.004 |
| Mean SDMT score at DMT initiation (SD) | | 50.5±12.5 | 50.8±12.8 | 0.763 |

DMT=disease-modifying therapy; EDSS=Expanded Disability Status Scale; SDMT=Symbol Digit Modalities Test; SD=standard deviation.

eTable 4. Baseline characteristics of participants included in the analysis of SDMT trajectories before and after escalation from platform to high-efficacy therapy.

| Characteristic | | Maintaining platform therapy | Escalation to high-efficacy DMT | P value |
| --- | --- | --- | --- | --- |
| N | | 362 | 3239 |  |
| Age at disease onset, years (SD) | | 36.0±11.2 | 30.6±9.3 | <0.001 |
| Age at time 0, years (SD) | | 50.5±11.7 | 41.0±10.5 | <0.001 |
| Years between onset and time 0 (SD) | | 14.9±9.9 | 10.6±7.8 | <0.001 |
| University, n (%) | Yes | 153 (42.3) | 1200 (37.0) | 0.450 |
|  | No | 179 (49.4) | 1520 (46.9) |  |
|  | Missing | 30 (8.3) | 519 (16.0) |  |
| Sex (%) | Female | 251 (69.3) | 2367 (73.1) | 0.13 |
|  | Male | 111 (30.7) | 872 (26.9) |  |
| Ancestry (%) | Nordic | 303 (85.4) | 2702 (83.9) | 0.49 |
|  | Non-Nordic | 52 (14.6) | 517 (16.1) |  |
| Mean baseline EDSS at time 0 (SD) | | 2.1±1.6 | 2.5±1.8 | 0.004 |
| Mean SDMT score at time 0 (SD) | | 50.5±10.8 | 50.1±12.5 | 0.68 |

SDMT=Symbol Digit Modalities Test; DMT=disease-modifying therapy; EDSS=Expanded Disability Status Scale; SD=standard deviation.

eTable 5. β coefficients and 95% CIs for SDMT trajectories over eight years after DMT initiation, by efficacy category.

| DMT efficacy category | β (95% CI)^1^ | P value | β (95% CI)^1-2^ | P value |
| --- | --- | --- | --- | --- |
| Platform therapy | Reference |  | Reference |  |
| High-efficacy therapy | 3.29 (2, 4.57) | <.0001 | 2.07 (0.66, 3.47) | 0.004 |
| Time | 1.46 (0.94, 1.97) | <.0001 | 1.17 (0.63, 1.7) | <0.0001 |
| Time 2 | -0.36 (-0.51, -0.21) | <.0001 | -0.29 (-0.46, -0.12) | 0.0008 |
| Time × DMT efficacy category | | | | |
| Platform therapy | Reference |  | Reference |  |
| High-efficacy therapy | 0.54 (0.27, 0.81) | <.0001 | 0.10 (-0.19, 0.39) | 0.50 |
| Time^2^ × DMT efficacy category | | | | |
| Platform therapy | Reference |  | Reference |  |
| High-efficacy therapy | -0.27 (-0.35, -0.19) | <.0001 | -0.27 (-0.37, -0.18) | <0.0001 |

SDMT=Symbol Digit Modalities Test; DMT=disease-modifying therapy; CI=confidence interval; EDSS=Expanded Disability Status Scale. ^1^adjusted for age at treatment initiation, sex, education, and the duration between onset and DMT initiation; ^2^adjusted for the number of repeated SDMT measurements.

eTable 6. Differences in SDMT scores between participants initiating platform versus high-efficacy DMT.

| Year | Platform therapy | High-efficacy therapy | Platform vs. high-efficacy DMT | |
| --- | --- | --- | --- | --- |
|  |  |  | Mean difference in SDMT (95% CI) | *P*-value |
| 0 | 425 | 709 | 0.70 (-1.30, 2.69) | 0.61 |
| 1 | 462 | 719 | -0.77 (-2.53, 0.99) | 0.51 |
| 2 | 422 | 642 | -1.69 (-3.40, 0.02) | **0.05** |
| 3 | 396 | 532 | -2.07 (-3.79, -0.34) | **0.01** |
| 4 | 364 | 430 | -1.89 (-3.67, -0.11) | **0.03** |
| 5 | 328 | 343 | -1.17 (-3.09, 0.74) | 0.29 |
| 6 | 292 | 267 | 0.10 (-2.14, 2.34) | 0.92 |
| 7 | 246 | 197 | 1.91 (-0.93, 4.76) | 0.24 |
| 8 | 169 | 133 | 4.27 (0.52, 8.03) | 0.02 |

The difference in estimated means was calculated as the mean Symbol Digit Modalities Test (SDMT) score among participants initiating platform therapy minus the mean SDMT score among those initiating high-efficacy DMTs. Thus, negative values indicate higher scores in participants starting on high-efficacy therapy. All estimates were adjusted for age at treatment initiation, sex, education, number of repeated SDMT measurements, and the duration between disease onset and treatment start.

eTable 7. β coefficient and 95% CIs for SDMT trajectories over eight years, by time from disease onset to high-efficacy DMT initiation.

| Time between MS onset and DMT initiation | β (95% CI)^1^ | P value | β (95% CI)^1-2^ | P value |
| --- | --- | --- | --- | --- |
| 0-5 years | Reference |  | Reference |  |
| 5-10 years | -0.57 (-1.72, 0.59) | 0.3374 | -0.04 (-1.23, 1.16) | 0.95 |
| >10 years | -2.19 (-3.38, -1.01) | 0.0003 | -2.07 (-3.29, -0.85) | 0.001 |
| Time | 2.13 (1.77, 2.49) | <0.0001 | 0.58 (0.16, 1.01) | 0.007 |
| Time ^2^ | -0.27 (-0.3, -0.23) | <0.0001 | -0.52 (-0.64, -0.41) | <0.0001 |
| Time × duration between disease onset and DMT efficacy category | | | | |
| 0-5 years | Reference |  | Reference |  |
| 5-10 years | 0.06 (-0.16, 0.27) | 0.596 | 0.14 (-0.07, 0.36) | 0.19 |
| >10 years | -0.16 (-0.36, 0.05) | 0.130 | -0.06 (-0.27, 0.15) | 0.59 |
| Time ^2^ × DMT efficacy category | | | | |
| 0-5 years | Reference |  | Reference |  |
| 5-10 years | -0.03 (-0.09, 0.03) | 0.333 | -0.09 (-0.16, -0.02) | 0.02 |
| >10 years | 0.07 (0.01, 0.13) | 0.014 | 0.01 (-0.07, 0.08) | 0.89 |

SDMT=Symbol Digit Modalities Test; DMT=disease-modifying therapy; CI=confidence interval. EDSS=Expanded Disability Status Scale. ^1^adjusted for age at treatment initiation, sex, education, and the duration between onset and DMT initiation; ^2^adjusted for the number of repeated SDMT measurements.

eTable 8. β coefficient and 95% CIs for SDMT trajectories over eight years among participants initiating Natalizumab versus anti-CD20 therapy.

| Treatment | β (95% CI)^1^ | P value | β (95% CI)^1-2^ | P value |
| --- | --- | --- | --- | --- |
| Natalizumab | Reference |  | Reference |  |
| Anti-CD20 therapies | -4.09 (-6.26, -1.92) | 0.0001 | -3.73 (-6.16, -1.31) | 0.003 |
| Time | 2.02 (1.27, 2.78) | <0.0001 | 1.71 (0.74, 2.69) | 0.001 |
| Time ^2^ | -0.77 (-0.99, -0.56) | <0.0001 | -1.03 (-1.36, -0.70) | <0.0001 |
| Time × DMT efficacy category | | | | |
| Natalizumab | Reference |  | Reference |  |
| Anti-CD20 therapies | -1.30 (-1.73, -0.86) | <.0001 | -0.77 (-1.26, -0.27) | 0.002 |
| Time ^2^ × DMT efficacy category | | | | |
| Natalizumab | Reference |  | Reference |  |
| Anti-CD20 therapies | 0.21 (0.08, 0.34) | 0.002 | 0.38 (0.21, 0.55) | <0.0001 |

SDMT=Symbol Digit Modalities Test; CI=confidence interval. EDSS=Expanded Disability Status Scale. ^1^adjusted for age at treatment initiation, sex, education, and the duration between onset and DMT initiation; ^2^adjusted for the number of repeated SDMT measurements.

eTable 9. β coefficients and 95% CIs for SDMT trajectories before and after escalation to high-efficacy DMT, comparing participants who escalated from platform therapy with those who remained on platform therapy.

|  | β (95% CI)^1^ | P value | β (95% CI)^1-2^ | P value |
| --- | --- | --- | --- | --- |
| Time before T_0_ × Escalation | | | | |
| Maintaining platform therapy | Reference |  | Reference |  |
| Escalation to high-efficacy DMT | -0.36 (-1.48, 0.75) | 0.52 | -0.63 (-1.75, 0.49) | 0.27 |
| Time^2^ before T_0_ × Escalation | | | | |
| Maintaining platform therapy | Reference |  | Reference |  |
| Escalation to high-efficacy DMT | 0.03 (-0.21, 0.27) | 0.83 | 0.00 (-0.24, 0.24) | 0.99 |
| Time after T_0_ (among those who escalated) | 3.61 (3.24, 3.97) | <0.0001 | 2.79 (2.35, 3.24) | <0.0001 |
| Time^2^ after T_0_ (among those who escalated) | -0.24 (-0.26, -0.22) | <0.0001 | -0.25 (-0.27, -0.23) | <0.0001 |

SDMT=Symbol Digit Modalities Test; DMT=disease-modifying therapy; CI=confidence interval.

T_0_ represents the time of escalation. ^1^adjusted for age at treatment initiation, sex, education, and the duration between onset and DMT initiation; ^2^adjusted for the number of repeated SDMT measurements.

eTable 10. Sensitivity analysis limited to incident cases: β coefficients and 95% CIs for SDMT trajectories over eight years after DMT initiation, by efficacy category.

| DMT efficacy category | β (95% CI)^1^ | P value | β (95% CI)^1-2^ | P value | β (95% CI)^1-3^ | P value |
| --- | --- | --- | --- | --- | --- | --- |
| Platform therapy | Reference |  | Reference |  | Reference |  |
| High-efficacy therapy | 2.11 (0.79, 3.43) | 0.002 | 1.58 (0.15, 3) | 0.03 | 0.93 (-1.18, 3.04) | 0.39 |
| Time | 1.47 (0.97, 1.97) | <0.0001 | 1.19 (0.64, 1.74) | <0.0001 | 0.82 (-0.12, 1.76) | 0.09 |
| Time ^2^ | -0.33 (-0.5, -0.17) | 0.0001 | -0.33 (-0.5, -0.16) | 0.0001 | -0.58 (-0.87, -0.3) | <0.0001 |
| Time × DMT efficacy category | | | | | | |
| Platform therapy | Reference |  | Reference |  | Reference |  |
| High-efficacy therapy | 0.18 (-0.08, 0.44) | 0.17 | 1.58 (0.15, 3) | 0.03 | 0.93 (-1.18, 3.04) | 0.39 |
| Time ^2^ × DMT efficacy category | | | | | | |
| Platform therapy | Reference |  | Reference |  | Reference |  |
| High-efficacy therapy | -0.28 (-0.37, -0.19) | <0.0001 | -0.29 (-0.39, -0.2) | <0.0001 | -0.28 (-0.43, -0.13) | 0.0001 |

SDMT=Symbol Digit Modalities Test; DMT=disease-modifying therapy; CI=confidence interval. EDSS=Expanded Disability Status Scale; MSIS=Multiple Sclerosis Impact Scale; ^1^adjusted for age at treatment initiation, sex, education, and the duration between onset and DMT initiation; ^2^adjusted for the number of repeated SDMT measurements; ^3^adjusted for ancestry, past infectious mononucleosis, smoking status, alcohol consumption, obesity, sun exposure, physical activity, baseline EDSS and MSIS-29.

eTable 11. Sensitivity analysis limited to incident cases: β coefficient and 95% CIs for SDMT trajectories over eight years among participants initiating Natalizumab versus anti-CD20 therapy.

| Treatment | β (95% CI)^1^ | P value | β (95% CI)^1-2^ | P value | β (95% CI)^1-3^ | P value |
| --- | --- | --- | --- | --- | --- | --- |
| Natalizumab | Reference |  | Reference |  | Reference |  |
| Anti-CD20 therapies | -2.94 (-5.14, -0.75) | 0.009 | -3.92 (-6.32, -1.51) | 0.002 | -5.53 (-8.96, -2.11) | 0.002 |
| Time | 1.61 (0.89, 2.33) | <0.0001 | 1.46 (0.49, 2.44) | 0.003 | 1.67 (0.21, 3.13) | 0.026 |
| Time ^2^ | -0.73 (-0.98, -0.48) | <0.0001 | -1 (-1.34, -0.67) | <0.0001 | -1.3 (-1.79, -0.81) | <0.0001 |
| Time × treatment type | | | | | | |
| Natalizumab | Reference |  | Reference |  | Reference |  |
| Anti-CD20 therapies | -0.61 (-1.03, -0.19) | 0.005 | -0.52 (-1.03, -0.02) | 0.04 | -0.68 (-1.4, 0.04) | 0.064 |
| Time^2^ × treatment type | | | | | | |
| Natalizumab | Reference |  | Reference |  | Reference |  |
| Anti-CD20 therapies | 0.35 (0.2, 0.5) | <0.0001 | 0.46 (0.28, 0.64) | <0.0001 | 0.52 (0.27, 0.78) | <0.0001 |

SDMT=Symbol Digit Modalities Test; CI=confidence interval. EDSS=Expanded Disability Status Scale; MSIS=Multiple Sclerosis Impact Scale; ^1^adjusted for age at treatment initiation, sex, education, and the duration between onset and DMT initiation; ^2^adjusted for the number of repeated SDMT measurements; ^3^adjusted for ancestry, past infectious mononucleosis, smoking status, alcohol consumption, obesity, sun exposure, physical activity, baseline EDSS and MSIS-29.

eTable 12. β coefficients and 95% CIs for SDMT trajectories among participants initiating high-efficacy DMT initiation, by baseline SDMT score (<median vs >median)

| Baseline SDMT category | β (95% CI) | P value | |
| --- | --- | --- | --- |
| Baseline SDMT <52 | Reference |  | |
| Baseline SDMT ≥52 | 13.95 (12.24, 15.67) | <0.0001 | |
| Time | 1.87 (1.18, 2.55) | <0.0001 | |
| Time ^2^ | -0.79 (-1.03, -0.55) | <0.0001 | |
| Time × Baseline SDMT category | | | |
| Baseline SDMT <52 | Reference |  | |
| Baseline SDMT ≥52 | -0.74 (-1.11, -0.36) | 0.0001 |  |
| Time^2^ × Baseline SDMT category | | | |
| Baseline SDMT <52 | Reference |  | |
| Baseline SDMT ≥52 | -0.01 (-0.14, 0.13) | 0.92 |  |

SDMT=Symbol Digit Modalities Test; DMT=disease-modifying therapy; CI=confidence interval. EDSS=Expanded Disability Status Scale; adjusted for age at treatment initiation, sex, education, number of repeated SDMT measurements, and the duration between onset and DMT initiation.

eFigure 1. Flow chart of the study population

MS=multiple sclerosis; GEMS=Genes and Environment in MS; EIMS=Epidemiological Investigation of MS; IMSE=Immunomodulation and MS Epidemiology; DMT=disease-modifying treatment; SDMT=Symbol Digit Modalities Test. For all analyses, we restricted the sample to participants with at least two SDMT assessments within the relevant treatment period to ensure reliable estimation of cognitive trajectories.

eFigure 2. SDMT trajectories among participants initiating high-efficacy DMT, by baseline SDMT score (<median vs >median)


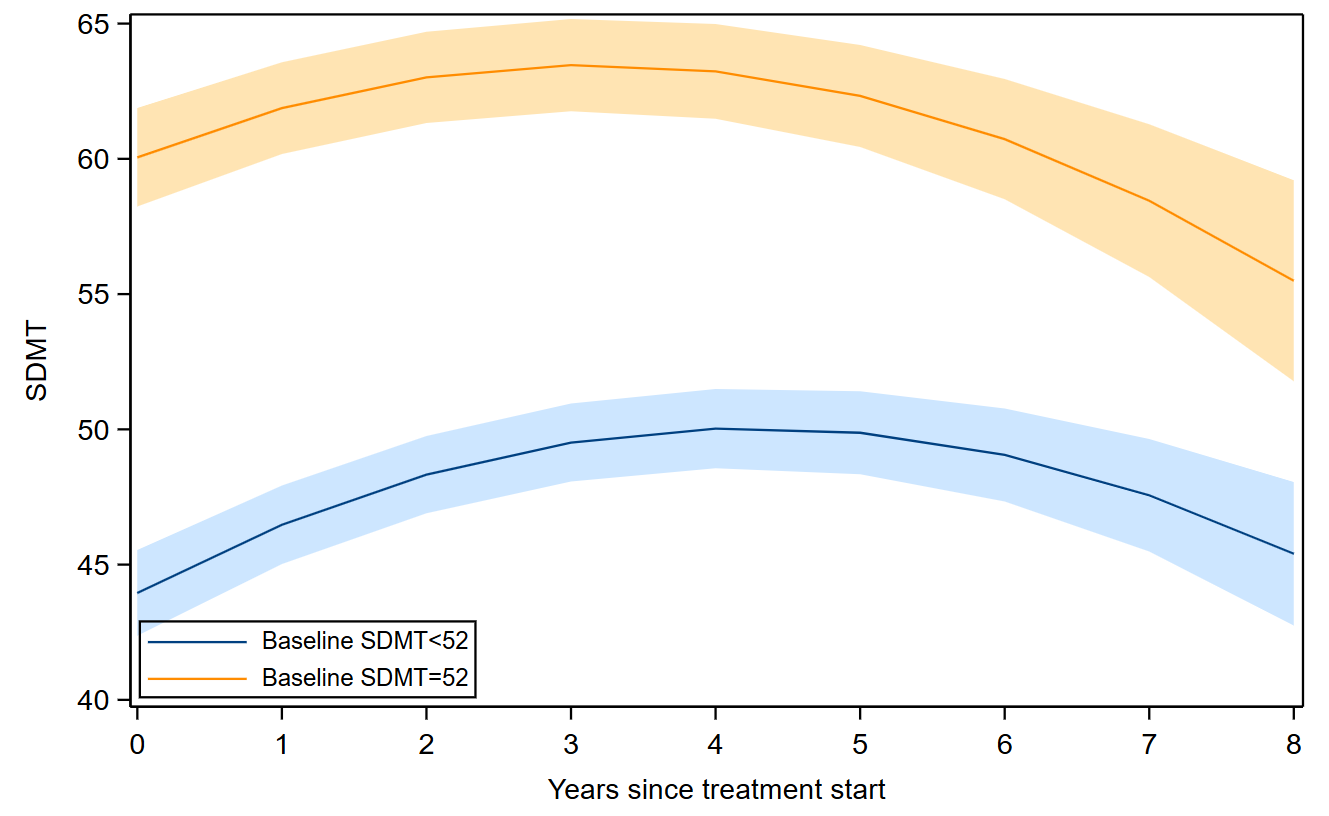


SDMT=Symbol Digit Modalities Test; DMT=high-efficacy disease-modifying therapy; adjusted for age at DMT initiation, sex, education, the repeated number of SDMT measurements, and time between onset and DMT initiation. The bands represent the 95% confidence interval for the estimated mean SDMT

eFigure 3. SDMT trajectories among DMT-naïve participants initiating platform versus high-efficacy DMT (incident cases)


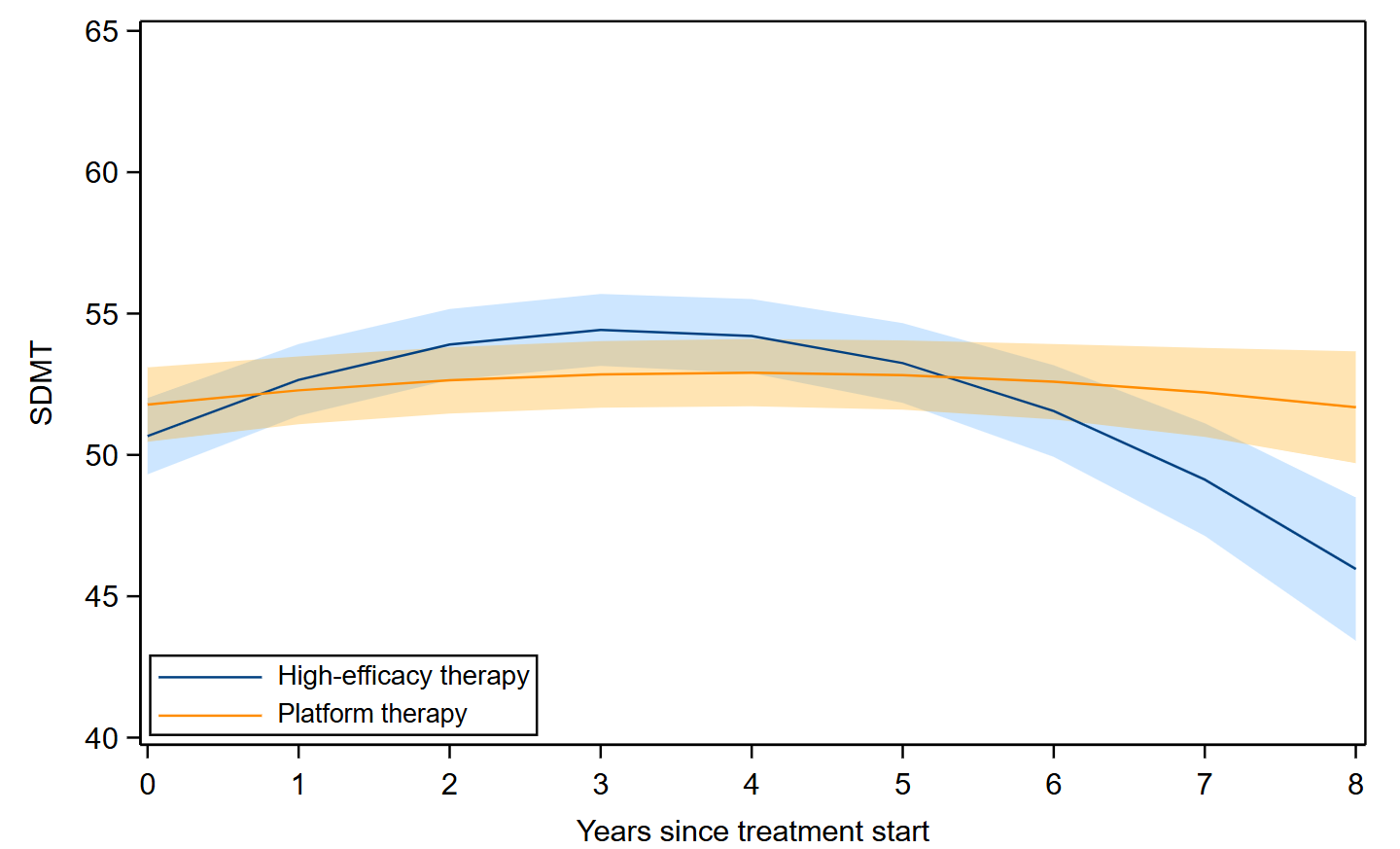


SDMT=Symbol Digit Modalities Test; DMT=high-efficacy disease-modifying therapy; adjusted for age at DMT initiation, sex, education, the number of repeated SDMT measurements, and the time between disease onset and DMT initiation. The bands represent the 95% confidence interval for the estimated mean SDMT.

eFigure 4. SDMT trajectories after initiation of Natalizumab versus anti-CD20 therapies (incident cases)


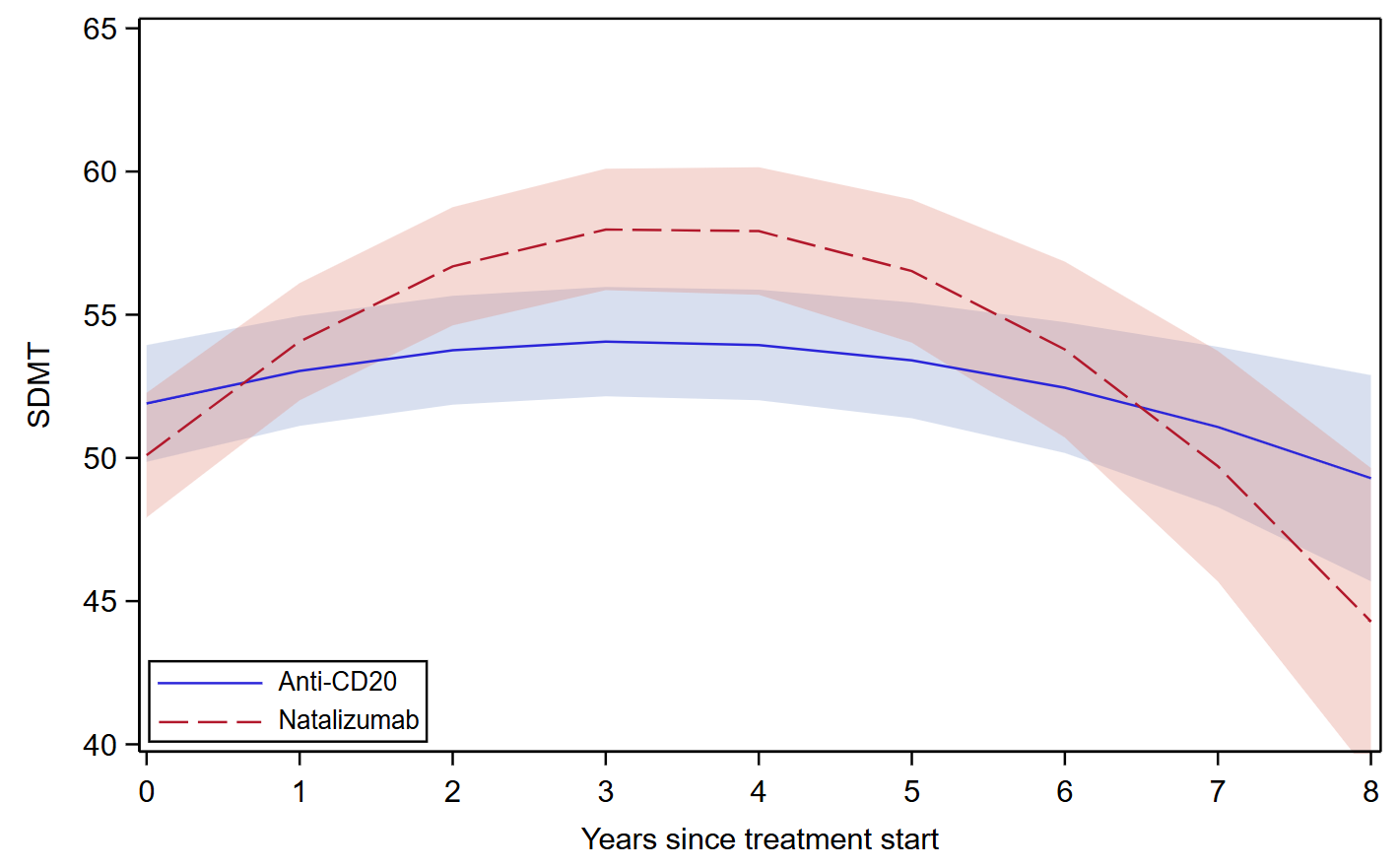


SDMT=Symbol Digit Modalities Test; DMT=high-efficacy disease-modifying therapy; adjusted for age at DMT initiation, sex, education, the number of repeated SDMT measurements, and time between disease onset and DMT initiation. The bands represent the 95% confidence intervals for the estimated mean SDMT.
